# Feasibility of intraoperative detection of sentinel lymph nodes with 89-zirconium-labelled nanocolloidal albumin PET-CT and a handheld high-energy gamma probe

**DOI:** 10.1186/s13550-018-0368-6

**Published:** 2018-02-14

**Authors:** Derrek A. Heuveling, K. Hakki Karagozoglu, Arthur Van Lingen, Otto S. Hoekstra, Guus A. M. S. Van Dongen, Remco De Bree

**Affiliations:** 1 Department of Otolaryngology - Head and Neck Surgery, VU University Medical Center, Amsterdam, The Netherlands; 2 Department of Oral and Maxillofacial Surgery/Oral Pathology, VU University Medical Center/Academic Center for Dentistry Amsterdam, Amsterdam, The Netherlands; 3 Department of Radiology and Nuclear Medicine, VU University Medical Center, Amsterdam, The Netherlands; 4 Department of Head and Neck Surgical Oncology, UMC Utrecht Cancer Center, University Medical Center Utrecht, University Utrecht, Heidelberglaan 100, 3584 CX Utrecht, P.O. Box 85500, 3508 GA Utrecht, The Netherlands

**Keywords:** Sentinel lymph node, Head and neck cancer, PET/CT lymphoscintigraphy, 89Zr-nanocolloidal albumin, High-energy gamma probe

## Abstract

**Background:**

PET/CT lymphoscintigraphy using ^89^Zr-nanocolloidal albumin has the potential to improve the preoperative identification of sentinel lymph nodes (SLNs), especially if located in the near proximity of the primary tumour. This study aims to demonstrate the feasibility of PET/CT lymphoscintigraphy followed by intraoperative detection of ^89^Zr-nanocolloidal albumin containing SLNs with the use of a handheld high-energy gamma probe.

**Methods:**

PET/CT lymphoscintigraphy was performed after peritumoural injection of ^89^Zr-nanocolloidal albumin in five patients with oral cavity carcinoma planned for surgical resection. SLN biopsy procedure was performed 18 h later. SLNs were detected using detailed information of PET/CT and the high-energy gamma probe.

**Results:**

In all patients, SLNs were identified on PET/CT lymphoscintigraphy. Intraoperative detection using the high-energy gamma probe was possible in 10 of 13 SLNs, at a short distance from the SLN.

**Conclusions:**

This study demonstrates that intraoperative detection of SLNs containing ^89^Zr-nanocolloidal albumin using a handheld high-energy gamma probe is feasible, but its clinical use and sensitivity seem to be limited.

**Trial registration:**

CCMO NL37222.092.11

## Background

For a successful sentinel lymph node (SLN) biopsy procedure, SLNs should correctly be identified and excised. Identification can be difficult when the SLN is located in close proximity of a primary tumour since this SLN can be hidden by the high amount of radioactivity at the injection site, the so-called ‘shine-through’ phenomenon. In such cases, the (limited) resolution of planar lymphoscintigraphy and SPECT/CT may hamper preoperative visualisation. For oral cancer, this is particularly true for primary tumours located in the floor of mouth (FOM) that drain to lingual and level I nodes, in which a lower sensitivity has been reported compared to other oral cavity subsites [1, 2].

Technical innovations to improve intraoperative SLN localisation include intraoperative real-time imaging, freehand SPECT and fluorescence imaging. Intraoperative real-time imaging with the portable gamma camera provides an overview of all radioactive spots and can show SLNs near the injection site by adjusting its position [3]. Freehand SPECT is designed to determine the position of the detector relative to the patient through which 3D images are generated. This provides the surgeon information about the direction and depth of the SLN in relation to the probe [4]. Another advantage of these intraoperative techniques may be the certainty it can provide about the completeness and accuracy of SN excision by showing the remaining activity. Near-infrared (NIR) fluorescence imaging may also be a very attractive option to facilitate intraoperative detection. NIR dyes have a limited but reasonable tissue penetration of excited and emitted light with negligible autofluorescence, resulting in higher target-to-background contrast. NIR fluorescence imaging provides high-resolution images which can be obtained in real time during the surgical procedure. Because of the limited penetration of NIR dyes like indocyanine green (ICG), NIR fluorescence imaging is usually combined with other techniques to navigate close to the SLN. The limited tissue penetration may also be an advantage since it results in negligible influence of fluorescence signal coming from the injection site [5]. Another suggested option to improve SLN procedure in FOM tumours is the dissection of the preglandular fat pad between the submandibular gland, the anterior belly of the digastric muscle, the mandible and the hyoid bone, the area where localisation of SLN may be hampered by the ‘shine-through’ phenomenon [6]*.*

Besides these potential intraoperative improvements, the need for better pre-operative visualisation of SLNs close to the injection site remains. A possible solution to solve this problem is the use of PET/CT for lymphoscintigraphy. PET/CT provides dynamic 3D information at a higher spatial resolution limiting the ‘shine-through’ effect and improves anatomic localisation—of particular importance in the complex anatomy of the neck with its abundant lymph nodes. Moreover, improved visualisation of lymphatic vessels may result in better differentiation between the first and second echelon lymph nodes. Recently, we demonstrated these advantages of SLN mapping using the PET tracer 89-zirconium (^89^Zr) labelled to nanocolloidal albumin (^89^Zr-nanocolloidal albumin) [7, 8]. However, intraoperative detection of ^89^Zr-nanocolloidal albumin containing SLNs has not been investigated yet.

The present study aimed to evaluate the feasibility of intraoperative detection of ^89^Zr-nanocolloidal albumin containing SLNs using a high-energy handheld high-energy gamma probe in patients with oral cavity carcinoma.

## Methods

### Patients

The study included five previously untreated patients with oral cavity carcinoma (maximum tumour size 4 cm) scheduled for surgical resection of the primary tumour including neck dissection, e.g. for patients with proven lymph node metastases (only clinically N1 disease) or patients in whom the neck needed to be opened for resection of the primary tumour or flap reconstruction. Tumour characteristics are summarised in Table 1. SLN biopsy was performed before the primary tumour was resected. The study was approved by the Medical Ethics Committee of the VU University Medical Center and the National Competent Authority. All patients gave written informed consent before inclusion.

**Table 1:** Results of PET/CT imaging and SLN procedure

Patient	Tumour	Clinical staging	Neck dissection levels dissected	Amount of ^89^Zr-nanocoll injected (MBq)	Number and level of the neck of SLN identified by PET/CT	Number and level of the neck of SLN intraoperatively detected by high-energy gamma probe	PA SLN	Final PA
1	Tongue L	T2N0	I–IV L	9	1 × IA L 1 × IB L 1 × III L 1 × III R	1 × IA L 1 × IB L 1 × III L 1 × III R	+ − −	T1N2b
2	Tongue R	T2N0	I–IV R	1	1× II R 1 × III R	n.d. n.d.		T2N2b
3	FOM L	T2N0	I–III L+R	2.4	2 × II/III L	1 × II L 2 × III L	− −	T1N0
4	Buccal mucosa L	T2N1	I–IV L	9	1 × II L	1 × II L	+	T2N2b
5	FOM R	T2N0	I–III L+R	8	1 × IB R 2 × II R 1 × III R	1 × IB R 2 × II R n.d.	− −	T1N0

*R* right side, *L* left side, *SLN* sentinel lymph node, *FOM* floor of mouth, *PA* histopathological result, *n.d.* not detected; *+* positive, *−* negative

As assessed by handheld high-energy gamma probe

### Preparation of ^89^Zr-nanocolloidal albumin

^89^Zr (*t*_1/2_ = 78.4 h) was provided by BV Cyclotron VU (Amsterdam, The Netherlands). ^89^Zr was conjugated to nanocolloidal albumin (Nanocoll; GE Healthcare, Eindhoven, The Netherlands) as described previously [3].

### Lymphoscintigraphy

One day prior to surgery, a PET/CT scan was performed after four peritumoural injection of on average a total of 5.9 ± 3.5 MBq of ^89^Zr-nanocolloidal albumin. The PET/CT scan (Gemini TF64; Philips Healthcare, Best, The Netherlands) consisted of three dynamic frames of 15 min each and was started almost directly after injection. After scanning, images were evaluated and the number and localisation (neck level) of SLNs were recorded. A clearly visible and rapidly (i.e. within the first frame) appearing lymph node was considered to be a SLN according to the definition of Morton [9]. Less visible lymph nodes (especially in the presence of a clear SLN) were considered the second echelon. All images were evaluated by a nuclear physician and head-and-neck surgeon, both experienced in SLN biopsy procedures in early-stage oral cancer patients.

### SLN biopsy procedure

**Fig. 1:**
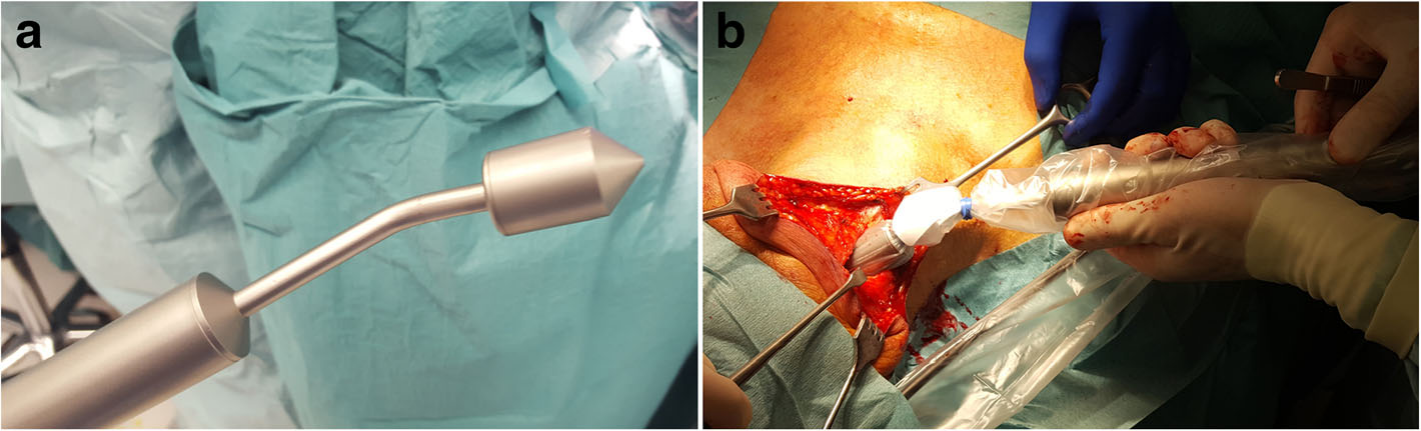
**a** High-energy gamma probe. **b** Intraoperative use of the handheld high-energy gamma probe

SLN biopsy was performed 18 h after injection of ^89^Zr-nanocolloidal albumin. Before skin incision, a handheld high-energy gamma probe (Gammalocater DXI; GF&E TEC GmbH; Seeheim, Germany) (Fig. 1), designed to detect 511-keV gamma rays, was used to identify the location of the SLN [10, 11]. Since the high-energy gamma probe uses five crystals with photodiodes to determine the direction of the incoming radiation, the probe head is rather large: approximately 3 cm in diameter and 6 cm long. This probe is in fact a multi-detector probe without mechanical collimation. Its focusing properties are achieved by parameterisation of the count rates from the different detectors inside the probe. Mechanical specifications are the following: probe handle of 20 cm, aperture detector of maximum 10 mm, outside diameter of maximum 30 mm and weight of 500 g. The performance of the probe is as follows (ref point: centre of and against aperture): counting sensitivity of > 10 cps/kBq, count rate range of 1–> 50 kcps, count rate linearity of < 10% loss up to 50 kcps, side shielding of < 0.1% and electronic collimation of < 50 mm FWHM at 50 mm distance (for comparison with a general low-energy probe, see Table 2).

**Table 2:** Comparison between high-energy probe and a general low-energy probe

	High-energy probe	Low-energy probe	Units
Source used	^22^Na	^99m^Tc	
Photon energy	511	142	keV
Sensitivity	10	12	cps/kBq
Spatial resolution			
@ 1 cm in air	15	10	mm
@ 5 cm in air	47	35	mm
Count rate range	1–50	1–100	kcps
Count rate loss	< 10%	< 10%	
Shielding	n.a.	1	mmPb
Weight	500	650	g

*n.a.* not available

During the surgical procedure, the area of interest was scanned with the high-energy gamma probe. An evident measurement of radioactivity by the probe, exceeding the background signal, was considered as a positive identification of a SLN. If the high-energy gamma probe did not identify the SLN at this point, the PET/CT information was used to localise the SLN. A skin incision was made in the incision line for the neck dissection. If the area of the SLN was reached, that particular region was scanned again by the high-energy gamma probe (Fig. 1b). After identification, the SLN was harvested. Ex vivo, the harvested lymph nodes were checked for radioactivity using the handheld high-energy gamma probe to confirm the presence of ^89^Zr-nanocolloidal albumin. In addition, the probe was used to check the neck for residual radioactivity.

Histopathological analysis (including step-serial sectioning and additional immunohistochemical staining using the pan-cytokeratin antibody AE 1/3) was performed on each excised SLN. After SLN biopsy, the neck dissection was completed and the surgical specimen was checked for remaining hot nodes.

Usage of the radionuclide ^89^Zr in the operating theatre has dosimetrical consequences for the personnel (Table 3). For the surgeon and personnel close to the patient (at 50 cm, 6 MBq administered), the dose rate amounts to 5 μSv/h. The dose to the surgeons’ hand for holding the radioactive specimen of 1 MBq/cm^3^ amounts to 0.4 mSv/s. No special precautions are taken on top of the normal operation theatre precautions (clothing etc.), except for collecting the surgeons’ gloves as radioactive waste.

**Table 3:** Dosimetrical aspects of ^89^Zr

Description		Units
Exposure rate	0.19	uSv/h/MBq m^2^
Re (inhalation)	3.5 × 10^9^	Bq
Hand dose	4 × 10^−10^	Sv/s per Bq/cm^2^
Half value thickness		
Lead	9	mm
Concrete	10.5	cm

## Results

**Fig. 2:**
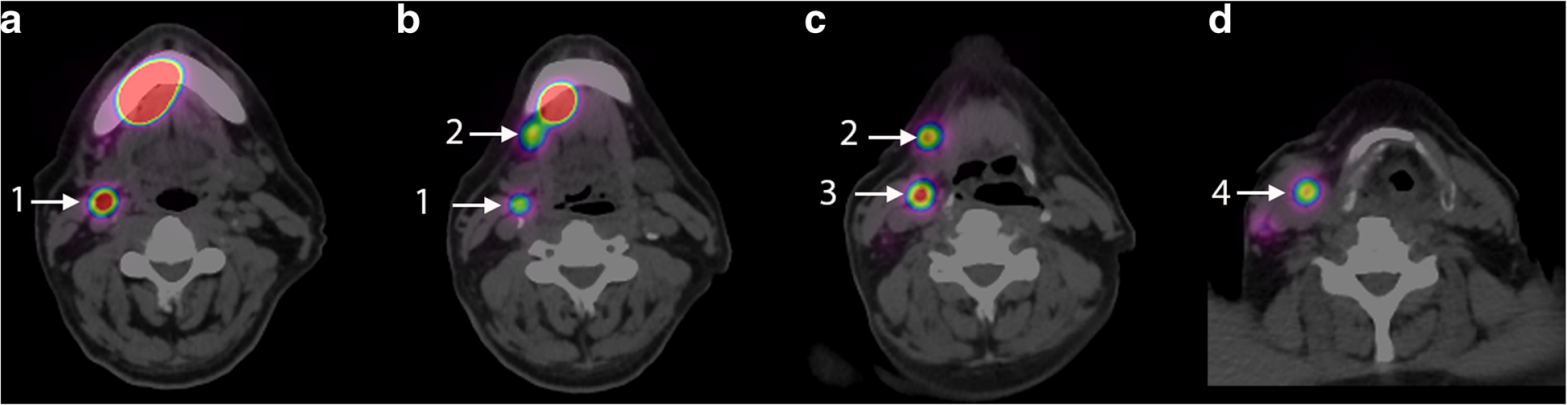
**a**–**d** Axial fused PET/CT images of patient 5 showing the injection site of ^89^Zr-nanocolloidal albumin in the floor of mouth on the right side and clear uptake in SLNs in levels IB (2), level II (1 and 3) and level III (4) on the right side. The distance between the injection site and the uptake in the SLN in level IB was 18 mm

Results of PET/CT and SLN biopsy procedure are summarised in Table 1. In all patients, at least one SLN was identified at PET/CT, some of which very close to the injection site (shortest distance 13 mm in patient 1 and 18 mm in patient 2 Fig. 2). Dynamic imaging appeared to be of no additional value in this (small) patient series, since all SLNs were already visible in the first frame. Lymphatic vessels towards SLN were visible in one patient.

**Fig. 3:**
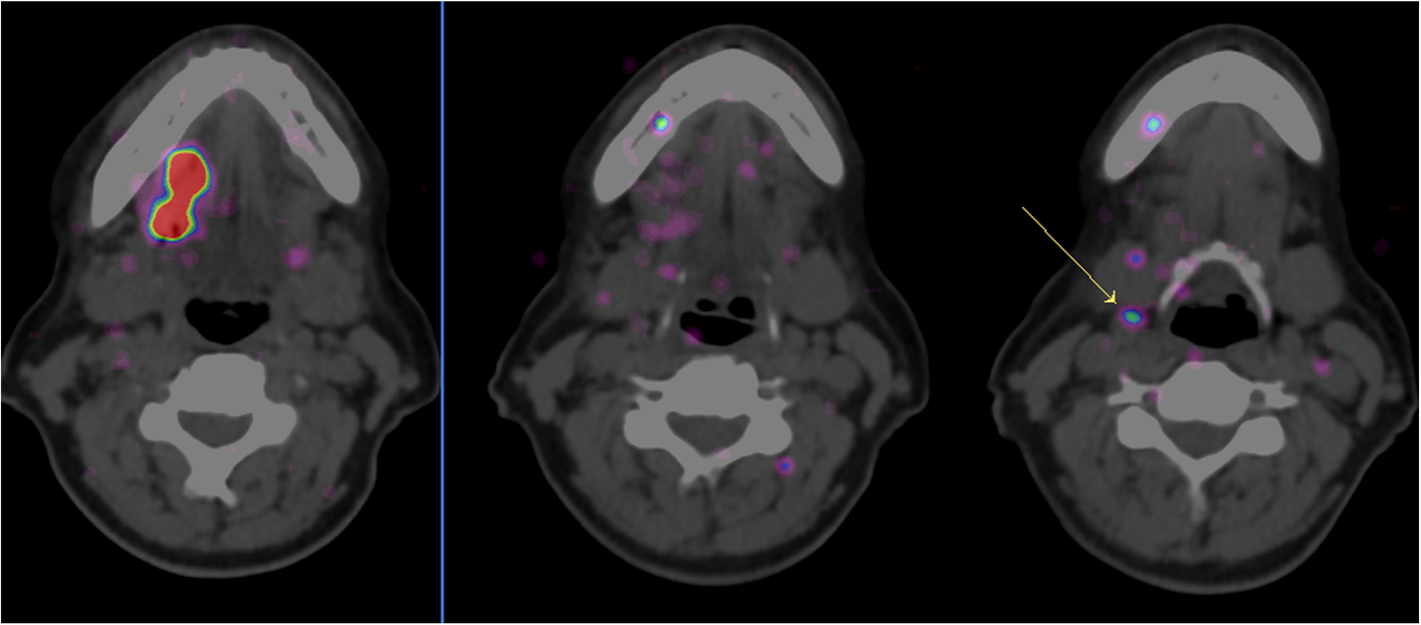
Axial fused PET/CT images of patient 2 showing the injection site of 89Zr-nanocolloidal albumin in the lateral tongue on the right side and low uptake in SLN in level II (arrow)

During the SLN biopsy procedure, we could not localise the SLNs with the high-energy gamma probe before elevation of the skin flaps. PET/CT images guided the surgeon to the approximate position of the SLN. Subsequently, the high-energy gamma probe pointed the exact localisation in 10 of 13 SLNs as defined on PET/CT. Preoperatively, the high-energy gamma probe was not easy to use due to its size as compared to a conventional gamma probe used for detection of 99m-technetium (^99m^Tc). After completion of the neck dissection, there were no additional hot nodes found in the neck dissection specimen. Three patients had N+ disease. In one patient (patient 2, Fig. 3), with the lowest dose administered, the positive SLN was not depicted by the high-energy gamma probe. None of the patients developed locoregional disease during follow-up (median follow-up 25 months, range 10–35 months).

## Discussion

Recently, we demonstrated the feasibility of PET/CT lymphoscintigraphy using ^89^Zr-nanocolloidal albumin as PET tracer for SLN detection. In the present study, we further investigated the feasibility of high-energy gamma probe-guided intraoperative detection of SLNs containing ^89^Zr-nanocolloidal albumin. The high-energy photon probe detected 10 of 13 SLNs as identified by PET/CT lymphoscintigraphy. Although the conventional use of ^99m^Tc-nanocolloidal albumin and SPECT/CT may result in the detection of additional SLN compared to gamma camera imaging, detection of level I lymph nodes in floor-of-mouth tumours remains challenging [12]. As demonstrated previously [8], PET/CT using ^89^Zr-nanocolloidal albumin was able to identify a SLN near the injection site.

Preoperative PET/CT was essential for guidance to the SLN because the high-energy gamma probe could not localise SLNs through the intact skin and was only useful in close proximity to the SLN. By using the combination of preoperative PET/CT imaging and intraoperative high-energy gamma probe, SLNs in level I of both patients with FOM could be harvested. The high-energy gamma probe failures were in patient 2 in whom the total amount of injected radioactivity was very low (≈ 1 MBq). Since the vast majority (probably > 95%) of the injected radioactivity will remain at the injection site, the amount of radioactivity in the SLN in this particular case was probably too low to allow detection with the high-energy gamma probe, although PET/CT was able to identify this SLN. The other failure (in patient 5) may be related to the same problem, in which a low uptake in the SLN in level III was observed on PET/CT (Fig. 2). The detection yield may improve when the optimal injected radioactivity dose for this high-energy gamma probe is used (i.e. a higher dose). Although this was not a dose-finding study, it might be expected that a dose of 8 MBq is high enough for SLN detection. Separate experiments with phantoms (unpublished data) learned that about 1 kBq of activity (under 1 cm of water) is needed for a count rate of 10 cps. With an internal counting time of 1 s, this will also be the amount of activity to be present in a patient before it can be detected.

A disadvantage of the high-energy photon high-energy gamma probe is its large size, which is due to the space needed for five crystals and photodiodes, elements that are necessary for a sensitive detection of the high-energy 511-keV photons. Due to its larger size, wider skin incision and exploration of the neck are needed. Another limitation of the high-energy gamma probe is that software takes care of how the ‘field of view’ is focused. The operator must be aware of the software settings chosen. In general, a surgeon is not very acquainted with this technical feature. Therefore, the design of the probe electronics and software should be adapted more to the surgical practice and the surgeon’s understanding of probes, in order to be more effective in localising the radioactive source. Because the high-energy photons travel a long distance in soft tissue, the high-energy gamma probe may still experience difficulties in the detection of a SLN which is in the proximity of the injection site. This study is the first study in which the novel PET tracer ^89^Zr-nanocolloidal albumin is used for preoperative lymphoscintigraphy and sentinel node biopsy. This study may serve as proof of concept for future studies on image-guided surgery using for example 89Zr-labelled monoclonal antibodies. As previously shown, PET/CT using ^89^Zr-nanocolloidal albumin may improve pre-operative visualisation of SLN but should probably be combined with other tracers (e.g. ICG) for intraoperative detection. Implementation of this PET approach may be hampered by the limited availability and high costs of PET imaging. The additional value of this technique should be weighed against these aspects for each indication. Intraoperative detection of SLNs using a handheld high-energy gamma probe appeared to be feasible but has obvious limitations. To our knowledge at the time of this project, there were only other probes that detect 511 keV radiation using thick and heavy collimators. The counting sensitivity of these probes was a factor 5–10 lower than our high-energy gamma probe. More suitable probes should be evaluated like for example the much smaller beta-probe, which detects directly the β^+^ particles [13]. These beta probes, however, are only effective when the probe is close to an active site (millimetre range). The results of this study should encourage the development of combined preoperative and intraoperative methods (e.g. a combination of PET-CT using a positron emitter-labelled tracer for preoperative PET-CT with a ^99m^Tc-labelled and/or ICG-labelled tracer for intraoperative detection) to improve the current SLN procedure.

## Conclusions

This study confirms the improved preoperative visualisation of SLNs using 89Zr-nanocolloidal albumin and demonstrates that intraoperative detection of SLNs containing 89Zr-nanocolloidal albumin using a handheld high-energy gamma probe is feasible, but its clinical use and sensitivity seem to be limited.
